# Breastmilk Production in the First 4 Weeks after Birth of Term Infants

**DOI:** 10.3390/nu8120756

**Published:** 2016-11-25

**Authors:** Jacqueline C. Kent, Hazel Gardner, Donna T. Geddes

**Affiliations:** School of Chemistry and Biochemistry, The University of Western Australia, Crawley 6009, Australia; Hazel.Gardner@uwa.edu.au (H.G.); Donna.Geddes@uwa.edu.au (D.T.G.)

**Keywords:** breastfeeding, milk production, insufficient milk

## Abstract

Breastmilk provides the ideal nutrition for the infant, and exclusive breastfeeding is recommended for the first 6 months. Adequate milk production by the mother is therefore critical, and early milk production has been shown to significantly affect milk production during established lactation. Previous studies indicate that milk production should reach the lower limit of normal for established lactation (440 mL per day) by day 11 after birth. We have used test-weighing of term infants before and after each breastfeed over 24 h to measure milk production in the first 4 weeks of lactation in mothers with and without perceived breastfeeding problems to provide information on how often milk production is inadequate. Between days 11 and 13, two-thirds of the mothers had a milk production of less than 440 mL per day, and between days 14 and 28, nearly one-third of the mothers had a milk production of less than 440 mL per day. The high frequency of inadequate milk production in early lactation and the consequence of suboptimal milk production in later lactation if left untreated suggest that objective measurement of milk production can identify mothers and infants at risk and support early intervention by a lactation specialist.

## 1. Introduction

There is a sound evidence base for the health advantages of breastfeeding for infants and their mothers, and a dose-response relationship has been demonstrated [1]. As such, any breastfeeding should be encouraged and full breastfeeding achieved if possible. Milk production and adequacy at 6 weeks after birth, for mothers of both healthy breastfeeding term infants and non-nursing preterm infants, have been shown to have a significant relationship with milk production 4–6 days after birth [2,3]. Hill et al. therefore suggest that interventions that promote an adequate milk supply by the first week postpartum are critical [2]. These include skin-to-skin contact and milk removal (breastfeeding or expressing) within an hour of birth and frequent milk removal during the first 24 h after birth. Early initiation of lactation, particularly breastfeeding or expressing within an hour of birth, has been shown to lead to a higher rate of breastfeeding beyond 6 weeks for term infants [4]. Skin-to-skin contact between mother and infant for the first hour after birth results in earlier effective breastfeeding [5] and an increased likelihood of breastfeeding 1–4 months after birth than when the infant was swaddled in blankets [6]. There is also a positive effect of the number of breastfeeds in the first 24 h on milk production on days 3 and 5 after birth [7]. It is important, therefore, to know if milk production is adequate during early lactation. The lower limit of normal daily milk production for established lactation has been calculated to be 440 g [8]. During the first week of lactation, on day 5, daily milk transfer during breastfeeding is 415 ± 123 g (combined data from a total of 305 breastfeeding mothers [7,9,10,11,12,13,14]). For mothers who were exclusively expressing breastmilk, one study of mothers of term infants measured a milk production of 973 ± 176 g per day on day 5 [15]. There is limited data on milk production during the second week after birth, but data from 10 mothers showed a daily milk transfer of 653 ± 154 g with a slightly higher milk yield of 668 ± 163 g due to some mothers occasionally pumping their breastmilk [11]. Hill et al. reported a lower milk production of 556 ± 187 g, but some of those mothers were supplementing [3]. Taken together, published data indicate that 92% of term mothers produce at least 440 g per day by 2 weeks of lactation.

It has been reported that 58% of term mothers experience problems with breastfeeding, including a perception of insufficient milk supply, in the first 2 weeks and this is associated with a lack of confidence in breastfeeding [16]. A perception of insufficient milk supply is given as a reason for the introduction of supplementary infant formula [17,18]. If breastmilk supply is actually inadequate supplementary feeds are necessary. If supplementary feeds are given instead of breastfeeds they could have a negative impact on milk supply. Measurement of milk production using in-home 24-h test-weighing is being used increasingly by lactation consultants and general practitioners as a clinical tool to either reassure mothers that their breastfeeding patterns and milk production are normal, or to guide advice to increase or decrease milk production or breastmilk transfer [19]. We aim to use 24-h milk profiles during the establishment of lactation to try to verify that full milk production is achieved by 2 weeks of lactation in mothers without perceived breastfeeding problems and, in a vulnerable population, how frequently milk supply is not adequate indicating that intervention would be appropriate.

## 2. Materials and Methods

A convenience sample of mothers within 4 weeks after the birth of a singleton, term infant (≥37 weeks gestational age at delivery, birth weight ≥2500 g) who were fully or partially breastfeeding [20] were invited to participate between June 2009 and April 2016. We recruited mothers under the care of general practitioners or lactation consultants for perceived breastfeeding problems, and volunteers for studies undertaken by the research group (e.g., Prime et al. [21]) who were without perceived breastfeeding problems. Demographics were recorded and the participants were loaned accurate digital scales (BabyWeigh™, Medela Inc., McHenry, IL, USA, resolution 2 g, accuracy ± 0.034%) to measure their milk profile. This involved the participants test-weighing their infants in their own homes [22] before and after each breastfeed or supplementary feed and recording amounts of breastmilk expressed. All measurements of breastfeed amounts and milk production are measured in grams but expressed in mL because the density of milk is 1.03 g·mL^−1^. Data were recorded either on paper or entered on a password-protected website accessed by invitation only. Breastfeeding parameters were calculated: the total amount of milk transferred from the mother to the infant while breastfeeding (total breastfeeding transfer), the amount of breastmilk expressed during the 24-h period (total breastmilk expressed), the total amount of milk produced by both breasts in the 24-h period (total breastmilk production = total breastfeeding transfer + total breastmilk expressed), and the total infant milk intake (total breastfeeding transfer plus expressed breastmilk and/or supplementary formula). The duration of each feed was taken from the time of weighing before to the time of weighing after the feeding.

Analysis used R version 3.2.1 GUI Snow Leopard (The R Foundation for Statistical Computing, Vienna, Austria) [23] with the base package and the library nlme [24] for linear mixed effects models. Summary statistics are presented as mean ± SD where the Shapiro-Wilk test indicated normality, or median (interquartile range) otherwise. Groups were compared on demographic and milk intake variables using two-tailed independent samples Student’s *t*-test where the Shapiro-Wilk test indicated normality, and Kruskal-Wallis rank sum test otherwise. Linear mixed effects analyses of the relationship between demographic and milk intake variables and total milk production were carried out with random effects of different intercepts for each mother. Differences were considered to be significant where *p* < 0.05. 

All participants supplied written, informed consent to participate in the studies, which were approved by the Human Research Ethics Committee at The University of Western Australia (RA/4/1/4103) and Women and Newborn Health Service (1746/ew).

## 3. Results

Sixty-two percent of participants who agreed to participate completed 24-h milk profiles between 6 and 28 days after birth. There were 13 participants without perceived breastfeeding problems (6 female infants, all Caucasian, 4 primiparous, 12 vaginal delivery) and 103 participants with perceived breastfeeding problems (50 female infants). Complete demographics were available for 48 participants (43 Caucasian, 32 primiparous, 28 vaginal delivery). Seventy-six of these provided information regarding their breastfeeding problems. The most common was a perception of insufficient milk supply (59 participants), but pain (11 participants) and positioning and attachment (10 participants) were also mentioned. Seventy-five of the participants with perceived breastfeeding problems were supplementing their infants with expressed breastmilk and/or infant formula, 45 using expressed breastmilk alone, 25 using infant formula and expressed breastmilk, and 5 participants used formula but no expressed breastmilk. The characteristics of the participants are presented in Table 1.

There were no significant differences between the groups for birth weight, frequency of breastfeeds, duration of breastfeeds, or total breastmilk production, but there were significant differences between the two groups for gestational age at delivery, average feed amount, total breastfeeding transfer, and total infant milk intake. Those with perceived breastfeeding problems who were supplementing with infant formula had a significantly lower average feed amount and total breastfeeding transfer (*p* < 0.001).

There were no significant relationships between infant age, birth weight, gestational age at delivery or average feed duration on total breastfeeding transfer (*p* > 0.13), but there were significant relationships between average feed duration and average feed amount (*R*^2^ = 0.08, *p* = 0.003), feed frequency and average feed amount (*R*^2^ = 0.10, *p* = 0.010), feed frequency and total breastfeeding transfer (*R*^2^ = 0.05, *p* = 0.018), and average feed amount and total breastfeeding transfer (*R*^2^ = 0.62, *p* < 0.001).

**Figure 1:**
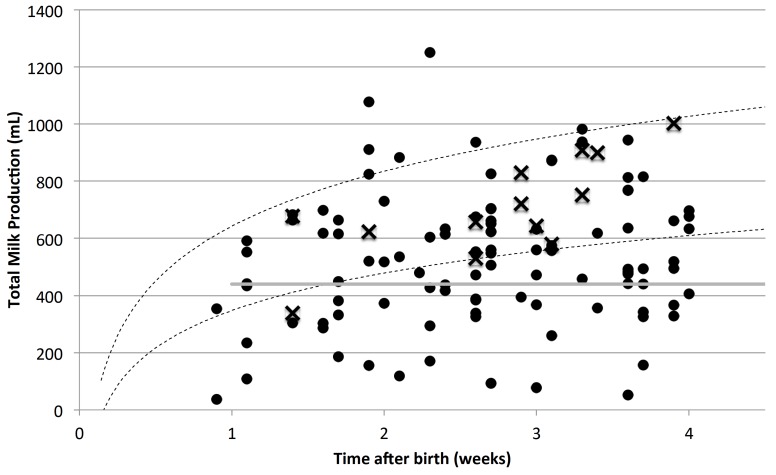
Total milk production of mothers with (circles) and without (crosses) perceived breastfeeding problems. The dotted lines indicate the mean ± SD of milk production derived from the literature [7,9,10,11,12,13,14,25]. The horizontal grey line indicates the lower limit of normal for established lactation [8].

All measurements of total milk production are shown in Figure 1. Twenty-nine measurements were made between 6 and 13 days of lactation. For those with perceived breastfeeding problems the total breastmilk production of 14 was ≥440 mL and 12 was <440 mL. Of the participants without perceived breastfeeding problems two had a total breastmilk production of 624 and 678 mL, and one who had a total breastmilk production of 338 mL at 1.4 weeks was an experienced breastfeeding mother (parity 4) who subsequently produced 722 mL at 5.1 weeks.

Eighty-seven measurements were made between 14 and 28 days of lactation. For the 77 of those with perceived breastfeeding problems, the total breastmilk production of 53 was ≥440 mL, and for 24 was <440 mL. All 10 participants without perceived breastfeeding problems had a total breastmilk production of ≥ 440 mL.

## 4. Discussion

The current data from all but one of the participants who did not have any perceived breastfeeding problems support the findings of limited published data suggesting that full milk production is normally reached by the second week of lactation. Even for mothers with perceived breastfeeding problems, over half were producing more than 440 mL per day in the first 13 days after birth.

It is normal for infants to lose weight after birth, and a recent study has demonstrated that the time of the nadir of weight occurs 52.3 h after birth with a loss of 218 g and a weight ratio (weight divided by birth weight) of 0.933 [26]. Compared with infants who were breastfed less than 7 times a day in the first 24 h after birth, infants who were breastfed 7 or more times received significantly more breastmilk, had a maximum weight loss that was 1% lower (5.8% compared with 6.8%), started to regain weight 19 h earlier, and had more than regained their birth weight by 7 days after birth [9]. Although there was no statistically significant difference in the feed frequency between those with and without breastfeeding problems, we observed that 11 of the participants with perceived breastfeeding problems had a feed frequency of <7, a mean total breastfeeding transfer of 195 mL, and a mean total breastmilk production of 344 mL. It is possible that this low production is a result of early infrequent breastfeeding. We suggest that if fully breastfed infants have not regained their birth weight by 7 days after birth the mother’s milk profile should be measured during the second week. If it is shown to be low (<440 mL) then early remedial action could be taken, which could include correction of positioning and attachment, increase in feeding frequency, and use of galactogogues. If an ample milk supply is demonstrated the infant should be assessed.

There were small effects of average feed duration and frequency on average feed amount, and a small effect of feed frequency on total breastfeeding transfer. However, the frequency and duration of breastfeeds for this population was very close to the average for mothers of exclusively breastfed infants between 4 and 26 weeks of lactation [17]. The most significant factor affecting total breastfeeding transfer was the average feed amount. This suggests that, while infants should be fed often and feed times should not be unnecessarily restricted, the major contributor to low total breastfeeding transfer is the amount of milk transferred during each breastfeed. About two-thirds of the mothers with perceived breastfeeding problems were already expressing breastmilk, suggesting that milk was possibly available but the infant was unable to remove sufficient milk during breastfeeding. The attention of the lactation specialist on good positioning and attachment and investigation of other possible reasons for poor milk transfer is reinforced by these data. The one participant who had a low measured milk production at 1.4 weeks gave only one extra breastfeed but doubled the amount transferred during each breastfeed and more than doubled her milk production by 5 weeks, illustrating that milk production can be increased during the early weeks as the infant becomes more effective at breastfeeding.

## 5. Study Limitations

Investigation of breastmilk production of mothers with and without breastfeeding problems in early lactation would benefit from a more detailed history of the initiation of lactation from the time of birth, including time of first breastfeed, skin-to-skin care, assessment of attachment, frequency of breastfeeds, and frequent measurements of the infants’ weights in the first week after birth. Measurement of 24-h milk profiles at 7 and 14 days after birth would allow identification of the factors that are most important in optimizing breastfeeding milk transfer. Weighing an infant before and after every feeding for 24 h can be demanding, so while this technique is very useful, it is not appropriate for all lactating mothers.

## 6. Conclusions

The high frequency of inadequate milk production in early lactation and the consequence of suboptimal milk production in later lactation if left untreated underline the importance of early identification of mothers and infants at risk. Measurement of milk profile during the second week of lactation provides an objective measure of breastfeeding milk transfer and total breastmilk production. These measures, considered with the infant’s weight changes, can provide an indication that early intervention by a lactation specialist to improve milk production is warranted.

## Figures and Tables

**Table 1:** Characteristics and breastfeeding parameters of participants without and with perceived breastfeeding problems.

	Perceived Breastfeeding Problems		*p* Value
	No	Yes	
*n*	13	103	
Birth weight (g)	3498 (293)	3450 (3206, 3739)	0.051
Gestational age at delivery (weeks^+days^)	39^+2^ (1^+3^)	39^+6^ (38^+7^, 40^+3^)	0.025
Feed frequency (breasts)	12 (3)	12 (4)	0.50
Average feed duration (min)	17 (5)	15 (13, 21)	0.052
Average feed amount (mL)	63 (27)	30 (20, 45)	<0.001
Total breastfeeding transfer (mL·day^−1^)	693 (174)	399 (211)	<0.001
Total breastmilk expressed (mL·day^−1^)	160 (*n* = 1)	168 (78, 272) (*n* = 68)	
Supplementary infant formula (mL·day^−1^)	0	135 (80, 272) (*n* = 72)	
Total infant milk intake (mL·day^−1^)	699 (168)	567 (164)	0.007

Data are presented as mean (SD) or median (IQR). Comparisons between measures for participants with and without perceived breastfeeding problems were made using Student’s *t*-test where the Shapiro-Wilk test indicated normality, and Kruskal-Wallis rank sum test otherwise.
